# Identification and Verification of Biomarker in Clear Cell Renal Cell Carcinoma via Bioinformatics and Neural Network Model

**DOI:** 10.1155/2020/6954793

**Published:** 2020-06-15

**Authors:** Bin Liu, Yu Xiao, Hao Li, Ai-li Zhang, Ling-bing Meng, Lu Feng, Zhi-hong Zhao, Xiao-chen Ni, Bo Fan, Xiao-yu Zhang, Shi-bin Zhao, Yi-bo Liu

**Affiliations:** ^1^Department of Urinary Surgery, The Fourth Hospital of Hebei Medical University, No. 12 Jiankang Road, 050000, China; ^2^School of Basic Medicine, Peking University, No. 38 Xueyuan Road, Haidian District, Beijing 100191, China; ^3^Department of Oncology, Dongzhimen Hospital, Beijing University of Chinese Medicine, Beijing, China; ^4^School of Basic Medical Sciences, Hebei Medical University, Shijiazhuang, Hebei, China; ^5^MOH Key Laboratory of Geriatrics, Beijing Hospital, National Center of Gerontology, Beijing, China; ^6^Department of Reproductive Medicine, The Fourth Hospital of Hebei Medical University, No. 12 Jiankang Road, 050000, China

## Abstract

**Background:**

Clear cell renal cell carcinoma (ccRCC) is the most common subtype of kidney cancer, which represents the 9th most frequently diagnosed cancer. However, the molecular mechanism of occurrence and development of ccRCC is indistinct. Therefore, the research aims to identify the hub biomarkers of ccRCC using numerous bioinformatics tools and functional experiments.

**Methods:**

The public data was downloaded from the Gene Expression Omnibus (GEO) database, and the differently expressed genes (DEGs) between ccRCC and normal renal tissues were identified with GEO2R. Protein-protein interaction (PPI) network of the DEGs was constructed, and hub genes were screened with cytoHubba. Then, ten ccRCC tumor samples and ten normal kidney tissues were obtained to verify the expression of hub genes with the RT-qPCR. Finally, the neural network model was constructed to verify the relationship among the genes.

**Results:**

A total of 251 DEGs and ten hub genes were identified. AURKB, CCNA2, TPX2, and NCAPG were highly expressed in ccRCC compared with renal tissue. With the increasing expression of AURKB, CCNA2, TPX2, and NCAPG, the pathological stage of ccRCC increased gradually (*P* < 0.05). Patients with high expression of AURKB, CCNA2, TPX2, and NCAPG have a poor overall survival. After the verification of RT-qPCR, the expression of hub genes was same as the public data. And there were strong correlations between the AURKB, CCNA2, TPX2, and NCAPG with the verification of the neural network model.

**Conclusion:**

After the identification and verification, AURKB, CCNA2, TPX2, and NCAPG might be related to the occurrence and malignant progression of ccRCC.

## 1. Introduction

Worldwide, renal cell carcinoma (RCC) represents the 9th most frequently diagnosed cancer in men and the 10th in women, accounting for 5% and 3% of all oncological diagnoses, respectively, [1]. According to the most updated data provided by the World Health Organization, there are more than 140 000 RCC-related deaths yearly, with RCC ranking as the 13th most common cause of cancer death worldwide. Age and gender factors are closely related to the risk of RCC. Other potential risk factors include lifestyle, complications, drugs, and environmental factors [2]. The diagnosis and management of RCC have changed remarkably rapidly in the past decades through the unremitting efforts of generation after generation of researchers. Despite progression in cancer control and survival, locally advanced disease and distant metastases are still diagnosed in a notable proportion of patients. Nevertheless, uncertainties, controversies, and research questions remain [3]. Further advances are expected from the diagnosis, treatment, and prognosis evaluation.

RCC is a group of heterogeneous tumors with different genetic and molecular changes, clear cell renal cell carcinoma (ccRCC), papillary RCC (type 1 and type 2), and chromophobe RCC are the most common solid RCC, accounting for 85.90% of all malignant RCC [4]. Among them, ccRCC is the most common subtype of kidney cancer. Both sporadic and inherited RCC are usually associated with structural changes in the short arm of chromosome 3 [5]. In addition, the occurrence of RCC is related to multiple gene alterations, such as VHL, PBRM1, BAP1, SETD2, TCEB1, and KDM5C [3]. Although our understanding of the biology of RCC has improved, surgery is still the main treatment method of RCC. Drugs and comprehensive therapies, identification of new target pathways, and optimal sequencing and combination of existing targeted drugs are areas that are worth researching [6].

Bioinformatics tools can screen differentially expressed genes (DEGs) between diseased and normal tissues [3, 7, 8]. These DEGs are related to the pathological stage, lesion grade, and prognosis of patients. Zou et al. used microarray technology to identify the hub genes between malignant glioblastoma and normal brain tissue and obtained the important targets related to brain glioma [9]. Through a series of bioinformatics analysis, Meng et al. concluded that TPM2 may be an important biomarker for the occurrence and development of atherosclerosis [10].

Therefore, this study will use bioinformatics technology to explore the gene molecular markers of abnormal expression during the occurrence of ccRCC and discuss the related potential mechanisms. These differentially expressed genes may affect the initiation and malignant progression of ccRCC and can be used as targets for diagnosis and treatment.

## 2. Material and Methods

### 2.1. Download Public Data

The Gene Expression Omnibus (GEO) database (http://www.ncbi.nlm.nih.gov/geo) is the largest, most comprehensive, and publicly available source of gene expression data.

On 20 December, 2019, we set key words “(clear cell renal cell carcinoma) AND (normal kidney)” to detect the datasets, using a filter of “expression profiling by array.” The inclusion criteria includes a diagnosis of clear cell renal cell carcinoma (data from papillary renal cell carcinoma diagnoses were excluded), the dataset including the gene expression profile of normal kidney (datasets which were composed of only tumor data were excluded), a sample number of more than forty per dataset (samples of less than forty were excluded), data from Homo sapiens (data from other species were excluded), and a series entry type, expression profiling by array (data using methylation profiling only by array were excluded).

Therefore, GSE105288 (GPL10558, Illumina HumanHT-12 V4.0 expression beadchip) and GSE66272 (GPL570(HG-U133_Plus_2) Affymetrix Human Genome U133 Plus 2.0 Array) were obtained from the GEO database. A total of 44 samples, including 35 ccRCC tissues and 9 normal renal tissues, were selected from GSE105288. A total of 53 samples, including 26 ccRCC tissues and 27 normal renal tissues, were selected from GSE66272.

### 2.2. Differentially Expressed Genes (DEGs) between Normal and PCRC

GEO2R (http://www.ncbi.nlm.nih.gov/geo/geo2r) could import data of the GEO database into the R language and perform differential analysis, essentially through the following two R packages, including limma packages and GEOquery. Therefore, through the GEO2R tool, DEGs were identified between the normal and ccRCC groups. The *P* values < 0.001 was defined as significant. The gene symbols were necessary. SangerBox (https://shengxin.ren), one open tool, was used to draw volcano maps. Venn diagrams were delineated using FunRich software (http://www.funrich.org/), which would visualize common DEGs shared between GSE105288 and GSE66272.

### 2.3. GO and KEGG Analysis

One online tool, DAVID (https://david.ncifcrf.gov/home.jsp) (version 6.8, Maryland, America), was applied to carry out the functional annotation for DEGs. Gene Ontology (GO) [11] generally performs enrichment analysis of genomes. And there are mainly cellular components (CC), biological processes (BP), and molecular functions (MF) in the GO analysis. Kyoto Encyclopedia of Genes and Genomes (KEGG) (https://www.kegg.jp/) [12] is a comprehensive database of genomic, chemical, and systemic functional information. Therefore, DAVID was used to make the analysis of GO and KEGG. The Biological Networks Gene Oncology tool (BiNGO) (version 3.0.3) was used to analyze and visualize the DEGs' cellular component, biological process, and molecular function [13].

### 2.4. Protein-Protein Interaction (PPI) Network

The common DEGs, shared between GSE105288 and GSE66272, were converted into differently expressed proteins. The STRING (Search Tool for the Retrieval of Interacting Genes) online database (http://string-db.org) could construct the PPI network, which was visualized by Cytoscape (version 2.8) [14].

### 2.5. Significant Module and Hub Genes

Molecular Complex Detection tool (MCODE) (version 1.5.1) [15], an open plug-in of Cytoscape, was performed to identify tested most significant module from the PPI network, and the criteria was that the maximum depth = 100, MCODE scores>5, cut − off = 2, *k* − score = 2, and node score cut − off = 0.2. Then, cytoHubba [16], a free plug-in of Cytoscape, was applied to authorize the hub genes, when the degree ≥ 10.

### 2.6. Expression Analysis of Hub Genes

The clustering analysis of expression level of hub genes was performed using heat maps based on the GSE105288 and GSE66272. Also, the expression profiles of hub genes in the ccRCC and normal groups were analyzed using Gene Expression Profiling Interactive Analysis (GEPIA, http://gepia.cancer-pku.cn/) [17].

### 2.7. Effect of Hub Gene Expression for Pathological Stage and Overall Survival

The effect of hub gene expression for pathological stage and overall survival was analyzed by the GEPIA. Finally, the correlation and linear regression analyses between AURKB, CCNA2, TPX2, and NCAPG were performed. And the receiver operator characteristic (ROC) curve analysis was performed to test the sensitivity and specificity of the hub gene expression for the diagnosis of ccRCC. The SPSS software (version 21.0; IBM; New York; America) was used to conduct all the statistical analysis. A *P* value < 0.05 was defined as statistically significant.

### 2.8. RT-qPCR Assay

A total of 10 ccRCC participates were recruited. After surgery, 10 ccRCC tumor samples from ccRCC patients and 10 adjacent normal kidney tissues samples were obtained. The research conformed to the Declaration of Helsinki and was authorized by the Human Ethics and Research Ethics Committees of the Fourth Hospital of Hebei Medical University. The informed consents were obtained from all participates.

Total RNA was extracted from 10 ccRCC tumor samples and 10 adjacent normal kidney tissue samples by the RNAiso Plus (TRIzol) kit (Thermo Fisher, Massachusetts, America and reverse transcribed to cDNA. RT-qPCR was performed using a Light Cycler® 4800 System with specific primers for genes.  Table 1 presents the primer sequences used in the experiments. The RQ values (2^−*ΔΔ*Ct^, where Ct is the threshold cycle) of each sample were calculated and are presented as fold change in the gene expression relative to the control group. GAPDH was used as an endogenous control.

### 2.9. The Confirmation Using The Cancer Genome Atlas (TCGA) Data

The gene expression dataset of ccRCC in the TCGA was downloaded using the University of California Santa Cruz (UCSC) Xena (https://xena.ucsc.edu/welcome-to-ucsc-xena/). There were a total of 944 samples including 537 ccRCC samples and 407 normal renal samples. The IlluminaHiSeq was selected as gene expression RNAseq in the research. In addition, the gene expression levels of VEGFA, AURKB, CCNA2, MCM2, MCM7, SMC4, TPX2, SLC2A1, MCM5, and NCAPG between ccRCC and normal renal samples were compared using the one-way ANOVA.

Furthermore, the effect of the gene expression of VEGFA, AURKB, CCNA2, MCM2, MCM7, SMC4, TPX2, SLC2A1, MCM5, and NCAPG on overall survival was analyzed by using the TCGA data.

### 2.10. The Construction of Neural Network Model

The training group was randomly divided into the calibration data and training data according to the proportion of 3 : 7. There were 6 samples in the calibration data, and 20 samples in the training data. We used MATLAB (version 8.3) to accomplish the normalization processing of variable values, network simulation, network training, and network initialization. The number of input neurons in the input layer is the same as the number of input variables, and the number is two. The hidden layer is designed as 1 layer, and the output layer is also designed for 1 layer. One output variable is the intima-media thickness. When training to 2000 steps after repeated training, the falling gradient is 0, and the training speed is uniform [10]. At the same time, the training error ≤ 0.05, and the *R* (relativity) value reached 0.9906.

## 3. Results

### 3.1. DEGs between Normal Kidney and ccRCC Samples

**Figure 1:**
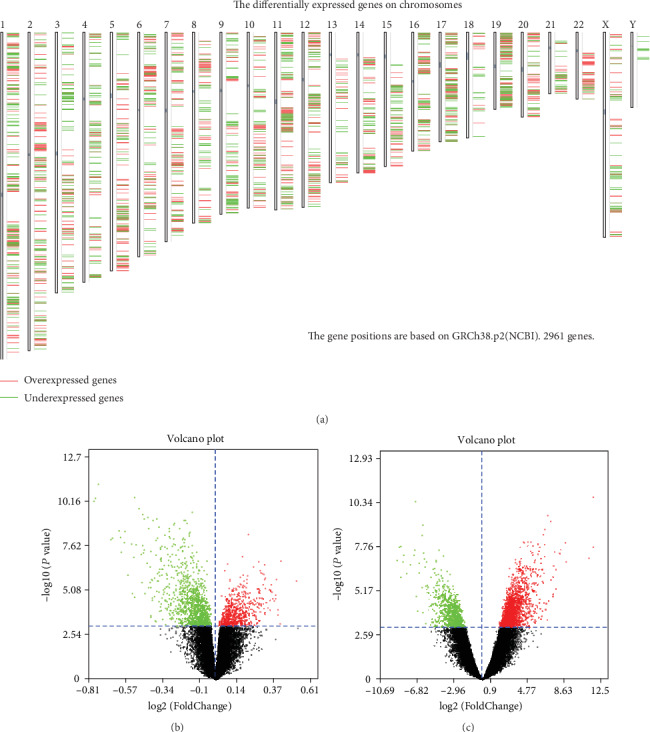
(a) The differentially expressed genes on chromosomes between ccRCC and normal kidney tissue. (b) One volcano plot presents the DEGs in the GSE105288. (c) Another volcano plot presents the DEGs in the GSE66272.

**Figure 2:**
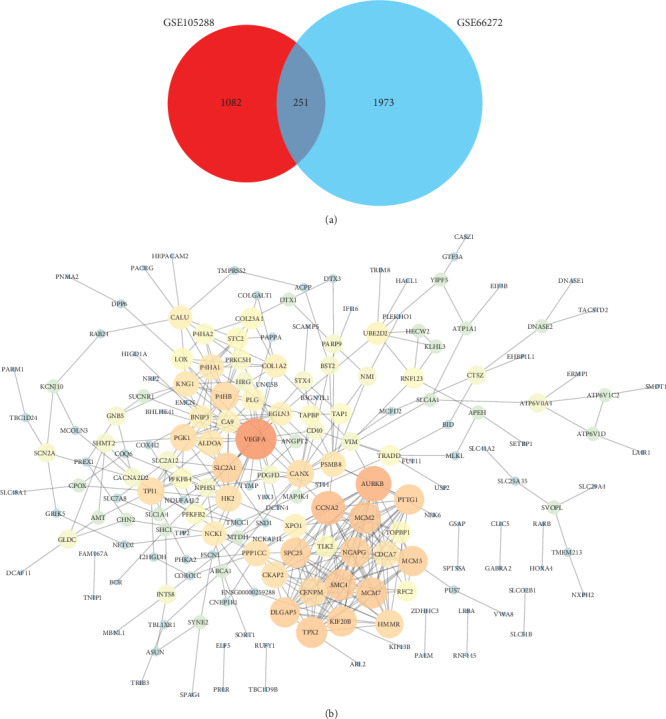
(a) The Venn diagram manifested that a total of 251 DEGs exist in the two datasets (GSE105288 and GSE66272) simultaneously. (b) The PPI network of the common DEGs.

There are plenty of DEGs on all chromosomes between the ccRCC and normal samples (Figure 1(a)). One volcano plot presents the DEGs in the GSE105288 (Figure 1(b)), and another volcano plot presents the DEGs in the GSE66272 (Figure 1(c)). The Venn diagram manifested that a total of 251 DEGs exist in the two datasets (GSE105288 and GSE66272) simultaneously (Figure 2(a)).

### 3.2. Construction of the PPI Network

After construction of the PPI network for the common DEGs, there are 189 nodes and 406 edges in the PPI network (Figure 2(b)).

### 3.3. The Functional Enrichment Analysis of DEGs via GO and KEGG

**Figure 3:**
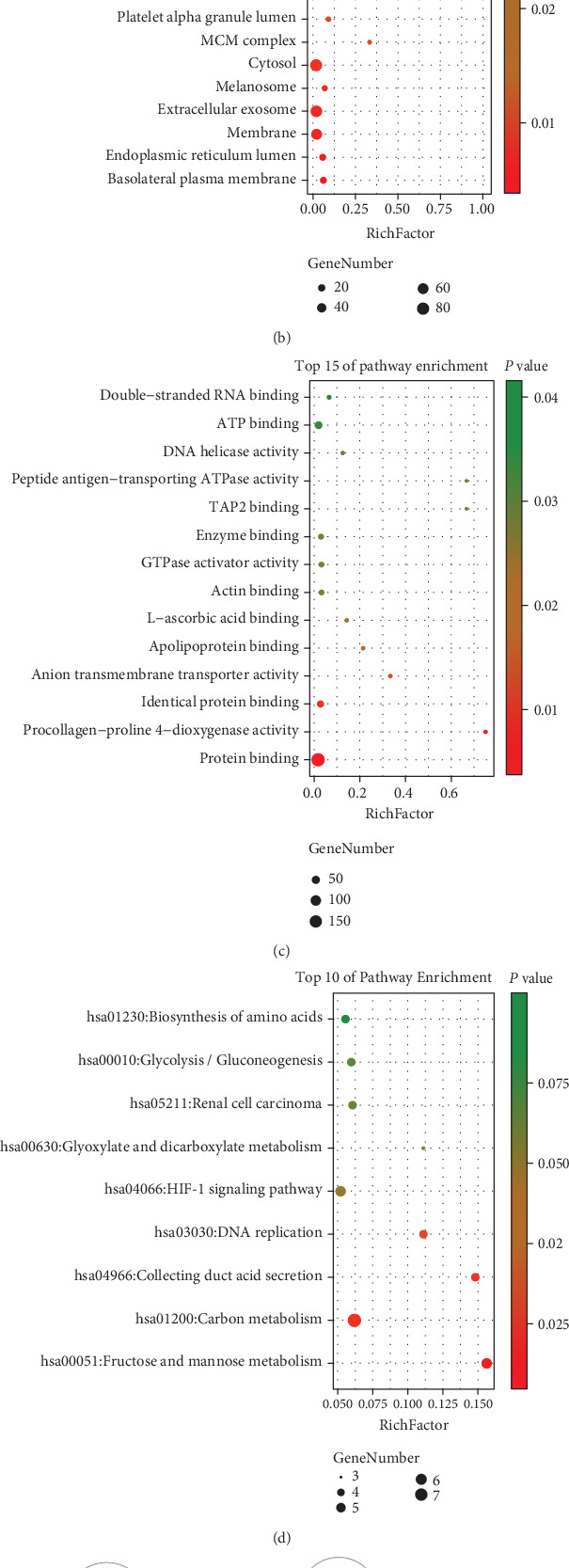
(a) Detailed information relating to changes in the biological processes (BP) of DEGs in ccRCC and normal kidney tissue. (b) Detailed information relating to changes in the cellular components (CC) of DEGs in ccRCC and normal kidney tissue. (c) Detailed information relating to changes in the molecular functions (MF) of DEGs in ccRCC and normal kidney tissue. (d) KEGG pathway analysis for DEGs. (e) The BP analysis for DEGs via the BiNGO software.

GO analysis manifested that variations in DEGs related with biological processes (BP) were significantly enriched in canonical glycolysis, glycolytic process, peptidyl-proline hydroxylation to 4-hydroxy-L-proline, angiogenesis, cell proliferation, fructose metabolic process, cell division, DNA replication initiation, regulation of insulin secretion, mitotic nuclear division, regulation of actin cytoskeleton organization, carbohydrate phosphorylation, glycine catabolic process, glycine decarboxylation via glycine cleavage system, cellular response to hypoxia, and so on (Figure 3(a)). The variations in DEGs related with cellular components (CC) were significantly enriched in the basolateral plasma membrane, endoplasmic reticulum lumen, membrane, extracellular exosome, melanosome, cytosol, MCM complex, and so on (Figure 3(b)). The variations in the DEGs related with molecular functions (MF) were significantly enriched in protein binding, procollagen-proline 4-dioxygenase activity, identical protein binding, anion transmembrane transporter activity, apolipoprotein binding, L-ascorbic acid binding, actin binding, and so on (Figure 3(c)). The KEGG pathway enrichment analysis showed that the top pathways related with DEGs were fructose and mannose metabolism, carbon metabolism, collecting duct acid secretion, DNA replication, HIF-1 signaling pathway, and so on (Figure 3(d)).

**Figure 4:**
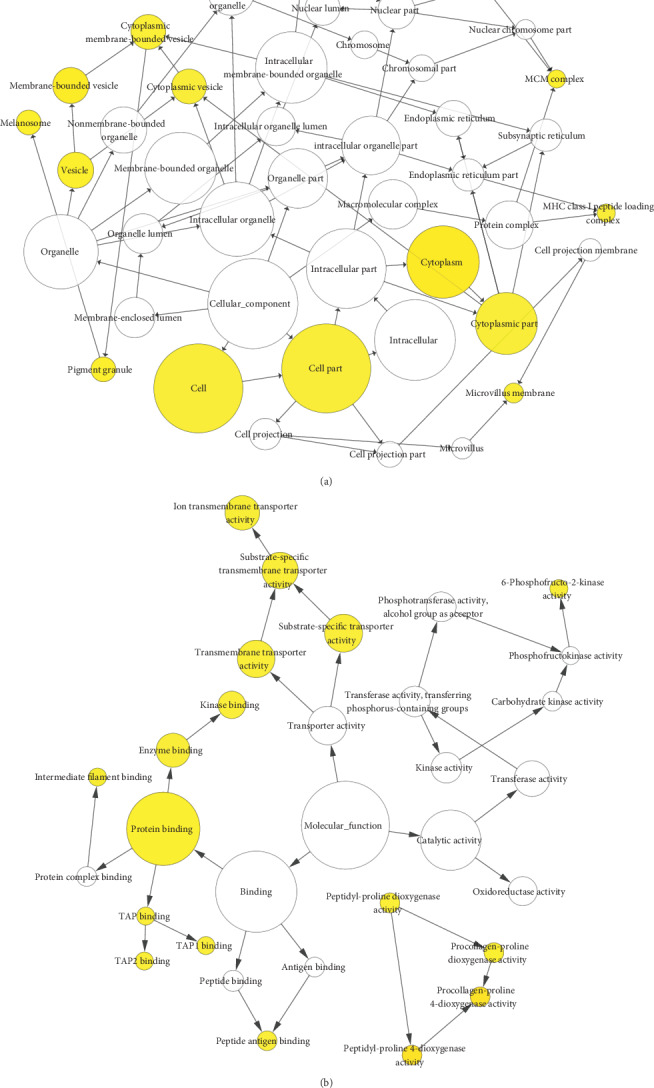
(a) The CC analysis for DEGs via the BiNGO software. (b) The MF analysis for DEGs via the BiNGO software.

The BP analysis for DEGs is presented in Figure 3(e) via the BiNGO software (Figure 3(e)). The CC analysis for DEGs is presented in Figure 4(a) via the BiNGO software (Figure 4(a)). The MF analysis for DEGs is presented in Figure 4(b) via the BiNGO software (Figure 4(b)).

### 3.4. Significant Module Network and Identification of Hub Genes

**Figure 5:**
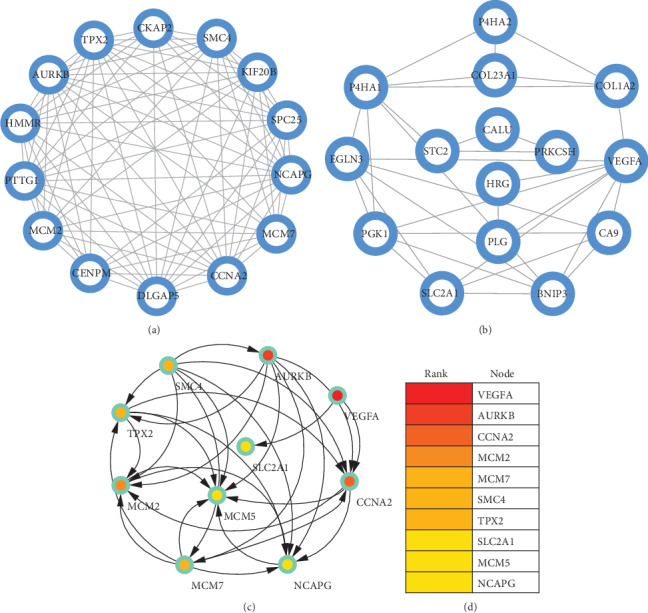
(a) A significant module was screened from the PPI network, and one module network consisted of 14 nodes and 84 edges. (b) Another module network consisted of 15 nodes and 32 edges. (c) Ten hub genes were identified, including VEGFA, AURKB, CCNA2, MCM2, MCM7, SMC4, TPX2, SLC2A1, MCM5, and NCAPG.

A significant module was screened from the PPI network, and one module network consisted of 14 nodes and 84 edges (Figure 5(a)). Another module network consisted of 15 nodes and 32 edges (Figure 5(b)). And ten hub genes were identified, including VEGFA, AURKB, CCNA2, MCM2, MCM7, SMC4, TPX2, SLC2A1, MCM5, and NCAPG (Figure 5(c)).

### 3.5. Difference of Expression of Hub Genes between ccRCC and Normal Kidney Samples

**Figure 6:**
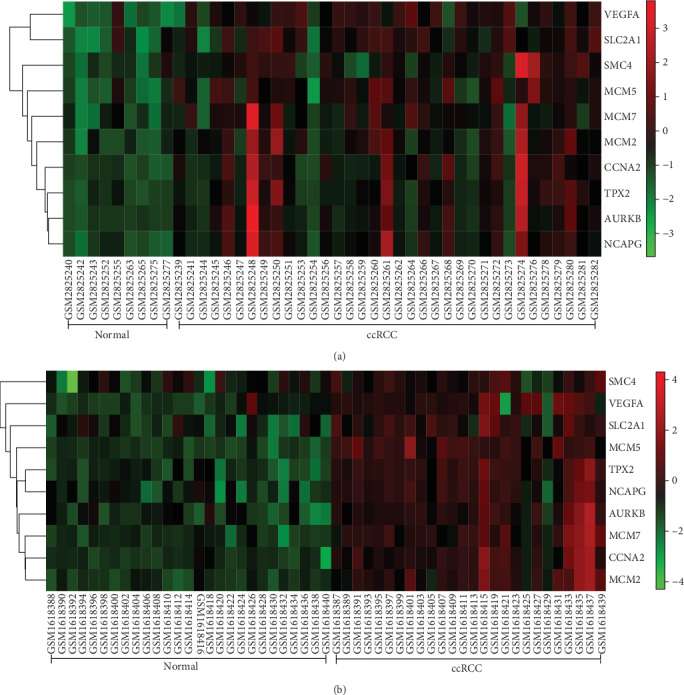
(a) One heat map showed that the expressions of all the hub genes were higher in the ccRCC samples than the normal samples in the GSE105288. (b) Another heat map also showed that the expressions of all the hub genes were higher in the ccRCC samples than the normal samples in the GSE66272.

**Figure 7:**
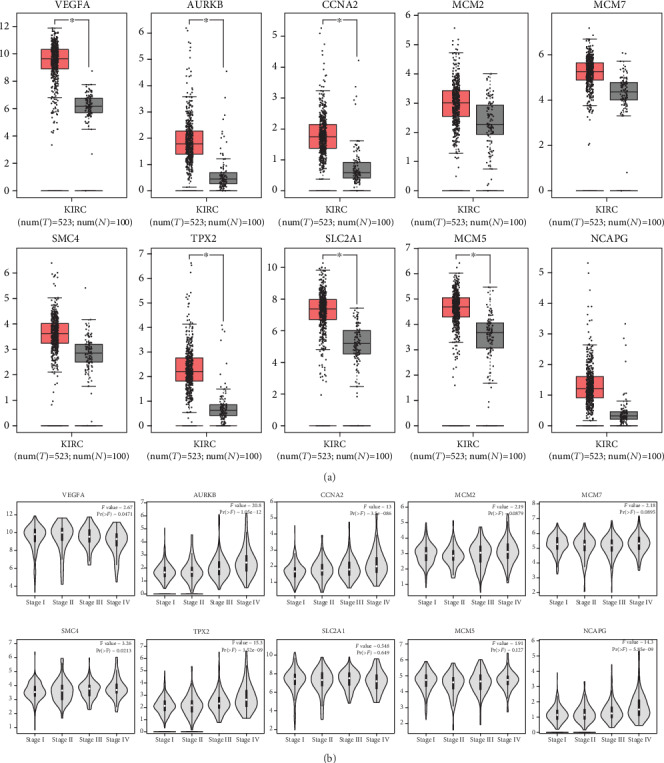
(a) The comparison of expressions of all hub genes between ccRCC and normal kidney samples. (b) The relationship between the expression of hub genes and pathological stage.

Hierarchical clustering allowed for simple differentiation of ccRCC tissues from normal colorectal tissues via the expression levels of hub genes in the GSE105288 and GSE66272 datasets. One heat map showed that the expressions of all the hub genes were higher in the ccRCC samples than the normal samples in the GSE105288 (Figure 6(a)). Another heat map also showed that the expressions of all the hub genes were higher in the ccRCC samples than the normal samples in the GSE66272 (Figure 6(b)). Through the GEPIA analysis, the expressions of hub genes in the ccRCC patients were higher than the normal individuals (Figure 7(a)).

### 3.6. Association between Hub Gene Expression, Pathological Stage, and Overall Survival

**Figure 8:**
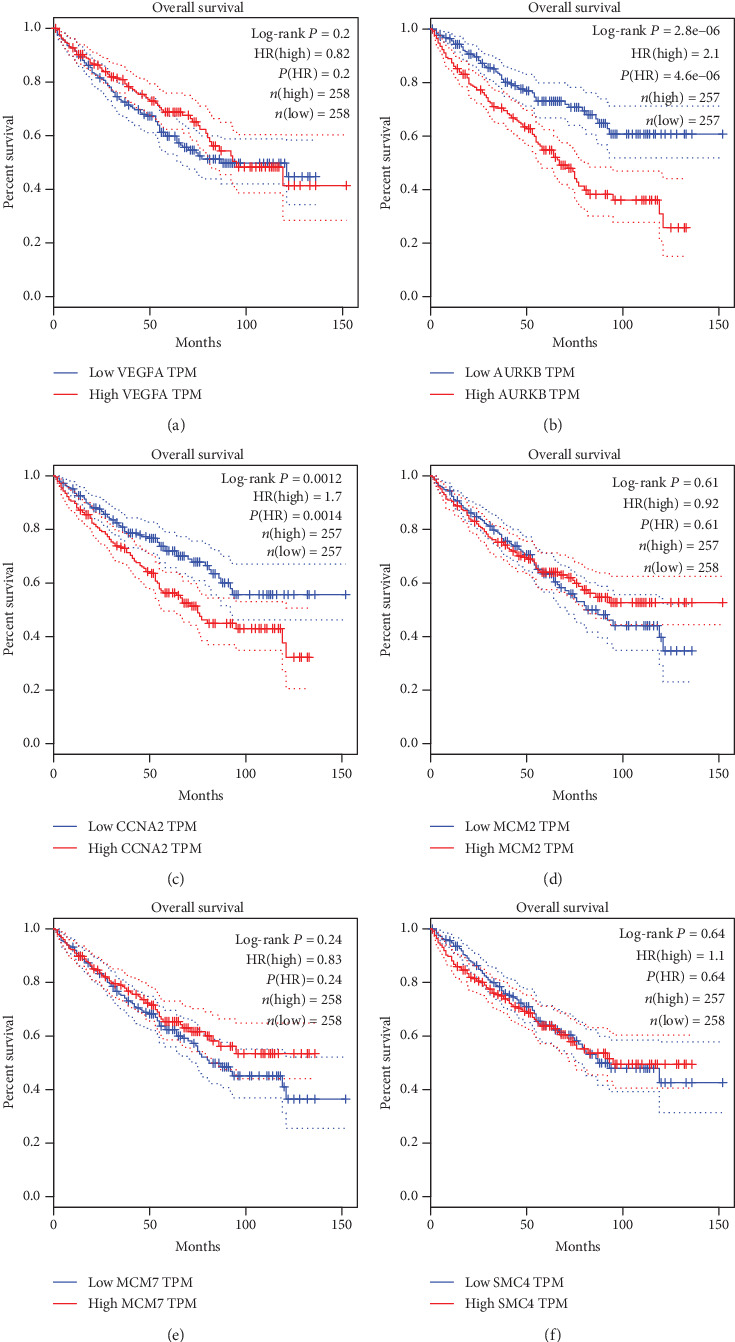
The overall survival Kaplan-Meier of six hub genes. (a) VEGFA, (b) AURKB, (c) CCNA2, (d) MCM2, (e) MCM7, and (f) SMC4.

**Figure 9:**
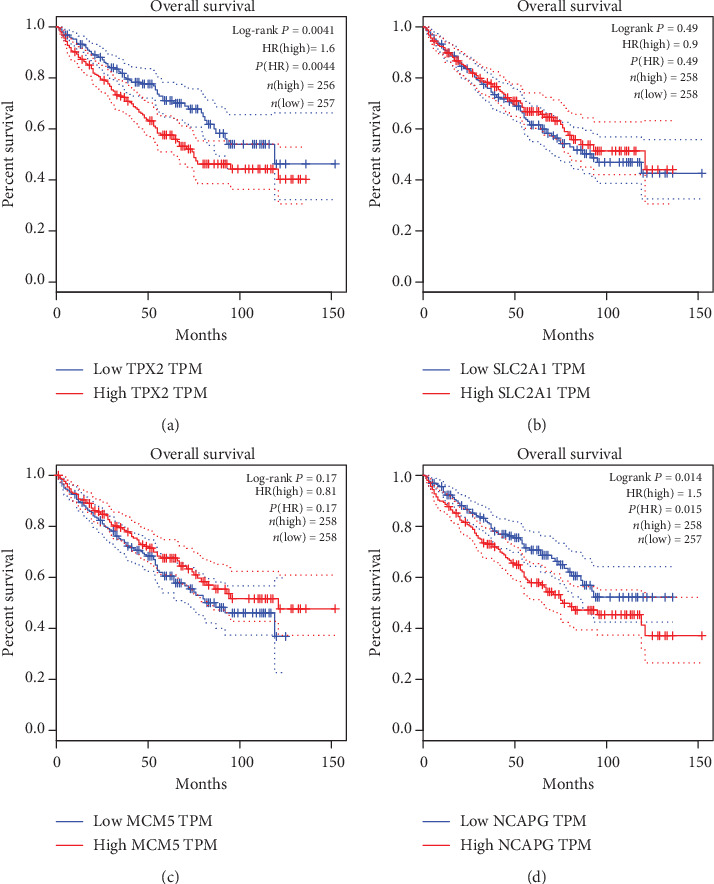
The overall survival Kaplan-Meier of another four hub genes. (a) TPX2, (b) SLC2A1, (c) MCM5, and (d) NCAPG.

The results of GEPIA manifested that the expressions of AURKB, CCNA2, TPX2, and NCAPG were significantly positively related with pathological stage (*P* < 0.05), while the expressions of VEGFA, MCM2, MCM7, SMC4, SLC2A1, and MCM5 were not (Figure 7(b)). The results showed that the expression level of VEGFA was not related with the overall survival of ccRCC patients (*P* > 0.05, Figure 8(a)). The overall survival analysis showed that ccRCC patients with high expression levels of AURKB (Figure 8(b)) and CCNA2 (Figure 8(c)) had poorer overall survival times than those with low expression levels (*P* < 0.05). The expression level of MCM2 was not related with the overall survival of ccRCC patients (*P* > 0.05, Figure 8(d)). The expression level of MCM7 was not related with the overall survival of ccRCC patients (*P* > 0.05, Figure 8(e)). The expression level of SMC4 was not related with the overall survival of ccRCC patients (*P* > 0.05, Figure 8(f)). The overall survival analysis showed that ccRCC patients with high expression levels of TPX2 had poorer overall survival times than those with low expression levels (*P* < 0.05, Figure 9(a)). The expression levels of SLC2A1 (Figure 9(b)) and MCM5 (Figure 9(c)) were not related with the overall survival of ccRCC patients (*P* > 0.05). The overall survival analysis showed that ccRCC patients with high expression levels of NCAPG had poorer overall survival times than those with low expression levels (*P* < 0.05, Figure 9(d)).

### 3.7. The Interaction Analysis among the Hub Genes

**Figure 10:**
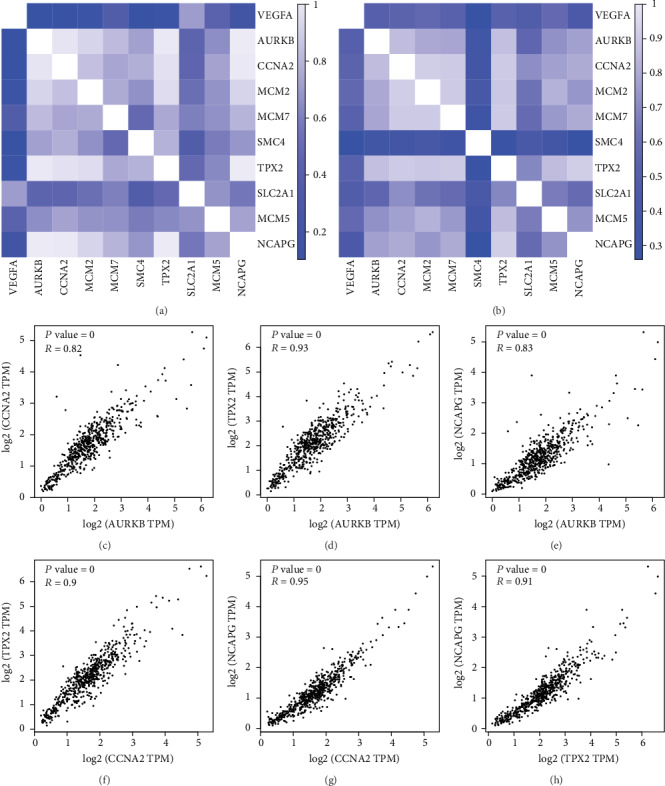
(a) Heat maps showing the correlations between hub genes in the GSE105288 datasets. (b) Heat maps showing the correlations between hub genes in the GSE66272 datasets. (c) The correlation between AURKB and CCNA2. (d) The correlation between AURKB and TPX2. (e) The correlation between AURKB and NCAPG. (f) The correlation between CCNA2 and TPX2. (f) The correlation between AURKB and NCAPG. (g) The correlation between CCNA2 and NCAPG. (h) The correlation between TPX2 and NCAPG.

Through the Pearson correlation test, heat maps manifested that there were strong correlations among hub genes in the GSE105288 (Figure 10(a)) and GSE66272 (Figure 10(b)) datasets. The correlation between AURKB, CCNA2, TPX2, and NCAPG was strong (Figures 10(c)–10(h)).

### 3.8. ROC Analysis

**Figure 11:**
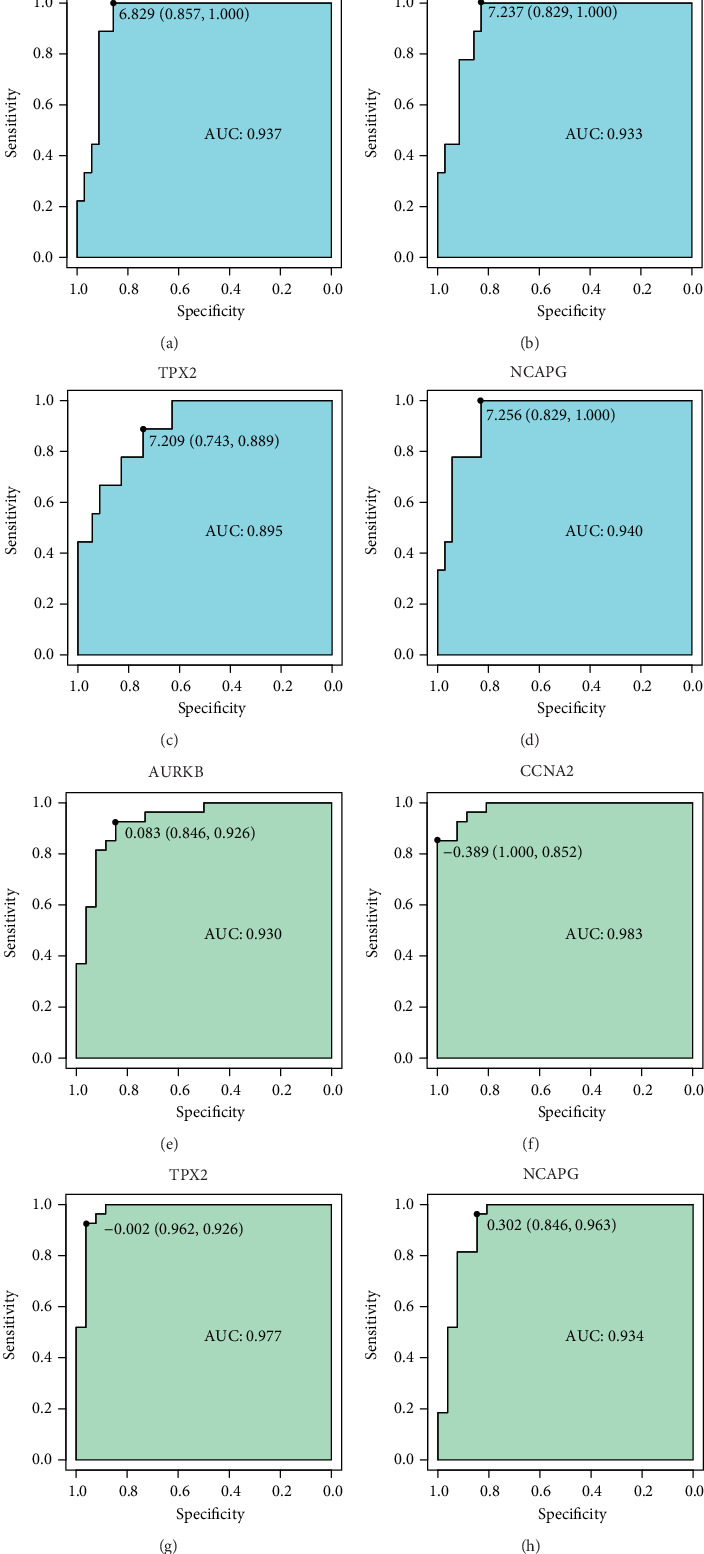
ROC curves of hub genes for ccRCC.

To identify accurate thresholds for hub genes to predict ccRCC, we constructed ROC. The expression of all hub genes was associated with a diagnosis of ccRCC. The ROC curve of AURKB in the GSE105288 was shown in Figure 11(a). The ROC curve of CCNA2 in the GSE105288 was shown in Figure 11(b). The ROC curve of TPX2 in the GSE105288 was shown in Figure 11(c). The ROC curve of NCAPG in the GSE105288 was shown in Figure 11(d). The ROC curve of AURKB in the GSE66272 was shown in Figure 11(e). The ROC curve of CCNA2 in the GSE66272 was shown in Figure 11(f). The ROC curve of TPX2 in the GSE66272 was shown in Figure 11(g). The ROC curve of NCAPG in the GSE66272 was shown in Figure 11(h).

### 3.9. Results of RT-qPCR Analysis

**Figure 12:**
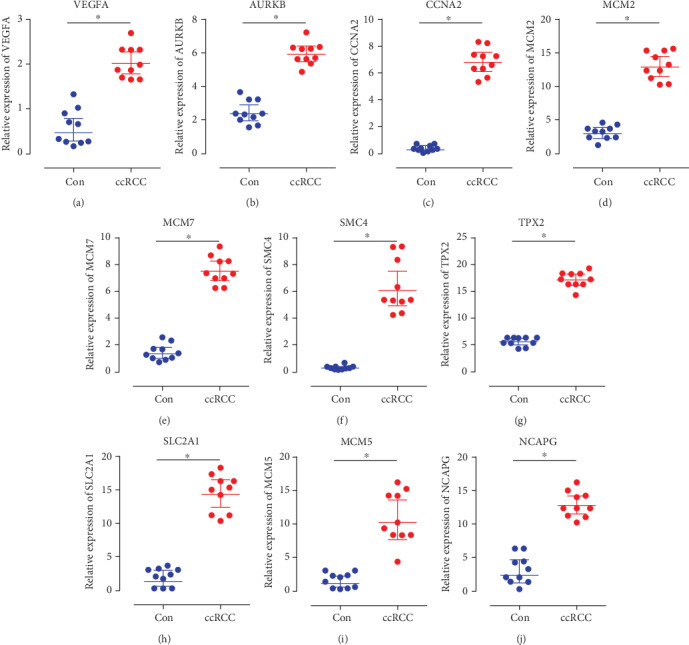
Relative expression of VEGFA, AURKB, CCNA2, MCM2, MCM7, SMC4, TPX2, SLC2A1, MCM5, and NCAPG by RT-qPCR analysis. ^∗^*P* < 0.05, compared with normal kidney tissues.

As presented in Figure 12, the relative expression levels of VEGFA, AURKB, CCNA2, MCM2, MCM7, SMC4, TPX2, SLC2A1, MCM5, and NCAPG were significantly higher in the ccRCC samples, compared with the normal kidney tissues groups. The result demonstrated that VEGFA, AURKB, CCNA2, MCM2, MCM7, SMC4, TPX2, SLC2A1, MCM5, and NCAPG might be considered biomarkers for ccRCC.

### 3.10. The Verification by TCGA

**Figure 13:**
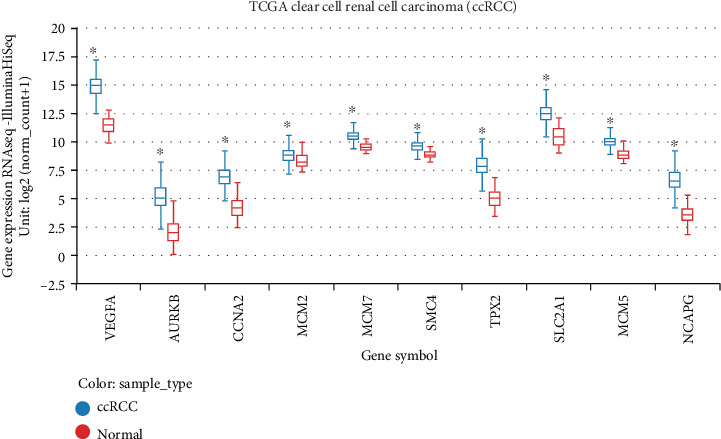
The confirmation of gene expression level using The Cancer Genome Atlas (TCGA) data. The gene expression levels of VEGFA, AURKB, CCNA2, MCM2, MCM7, SMC4, TPX2, SLC2A1, MCM5, and NCAPG in ccRCC samples were significantly higher than the normal renal samples.

According to the above expression analysis, VEGFA, AURKB, CCNA2, MCM2, MCM7, SMC4, TPX2, SLC2A1, MCM5, and NCAPG were markedly upregulated in ccRCC tumor samples compared with the normal renal samples. After confirmation using the TCGA data, these gene expression levels in the ccRCC samples were also significantly higher than the normal renal samples (*P* < 0.05, Figure 13).

**Figure 14:**
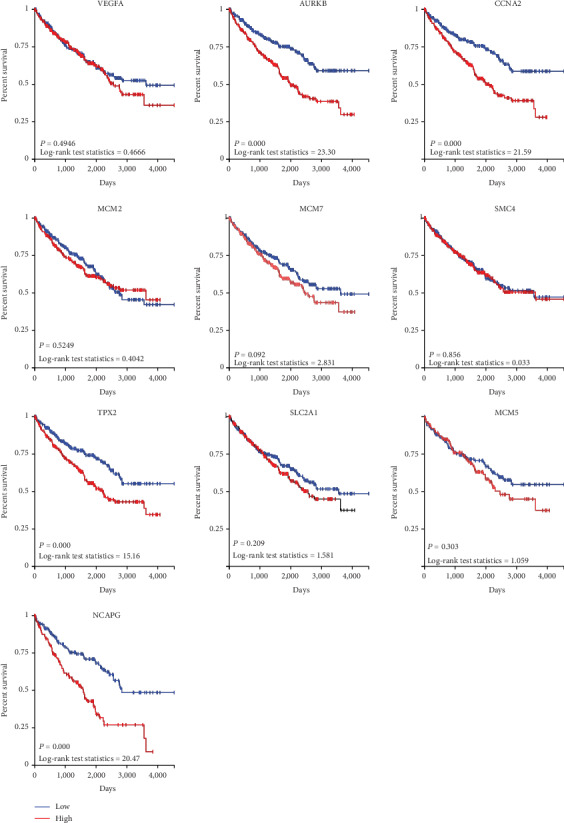
The effect of gene expression on overall survival by using the TCGA data. The expression level of VEGFA, MCM2, MCM7, SMC4, SLC2A1, and MCM5 was not related with the overall survival of ccRCC patients. The ccRCC patients with high expression levels of AURKB, CCNA2, TPX2, and NCAPG had poorer overall survival times than those with low expression levels.

The results showed that the expression level of VEGFA (*P* = 0.4946), MCM2 (*P* = 0.5249), MCM7 (*P* = 0.092), SMC4 (*P* = 0.856), SLC2A1 (*P* = 0.209), and MCM5 (*P* = 0.303) was not related with the overall survival of ccRCC patients. The overall survival analysis showed that ccRCC patients with high expression levels of AURKB (*P* = 0.000), CCNA2 (*P* = 0.000), TPX2 (*P* = 0.000), and NCAPG (*P* = 0.000) had poorer overall survival times than those with low expression levels (Figure 14).

### 3.11. The Neural Network Prediction Model between AURKB, CCNA2, TPX2, and NCAPG

**Figure 15:**
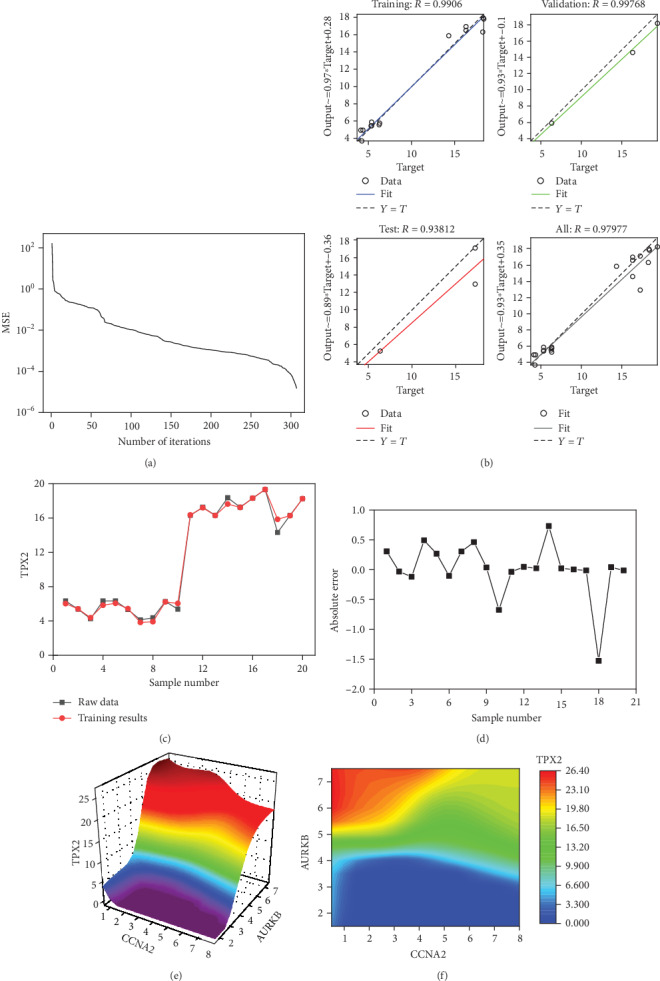
The neural network models. (a) The best training performance. (b) The relativity of training, validation, test, and all procedure. (c) The comparison chart of training results. (d) The error analysis diagram. (e) The high-risk warning range at the level of the three-dimensional stereogram. (f) The high-risk warning range at the level of the plane form.

The mean squared error is <0.05 (Figure 15(a)). The relativity of training is 0.9906. The relativity of validation is 0.99768. The relativity of test is 0.93812. And the relativity of all procedure is 0.97977 (Figure 15(b)). Through verifying the predicted value of the data against the actual value, we found that there are only small differences in the comparison chart of training results (Figure 15(c)) and error analysis diagram (Figure 15(d)). Based on the above result, we could speculate that there were strong correlations between AURKB, CCNA2, TPX2, and NCAPG.

Through the cubic spline interpolation algorithm, we find the high-risk warning indicator of TPX2: CCNA2 < 5.0 and 5.2 < AURKB. The three-dimensional stereogram could present the warning range well (Figure 15(e)). The plane graph is also shown (Figure 15(f)).

## 4. Discussion

RCC is a common disease in the urinary system. According to the statistics of the World Health Organization in 2018, its incidence is second only to prostate cancer and bladder cancer and is increasing year by year [1]. Although many genes are considered potential therapeutic targets and prognostic predictors of RCC, the molecular mechanism of the occurrence and development of RCC remains controversial.

With the continuous progress of science, microarray technology, as a special data mining method, is very influential at present. This revolutionary technology transforms traditional molecular research from a situation that relies on personal experience and subjective guesses to a more objective science [18–20].

In this paper, bioinformatics tools are used to mine the targeted biomarkers of ccRCC. The results showed that AURKB, CCNA2, TPX2, and NCAPG were highly expressed in ccRCC compared with renal tissue. With the increasing expression of AURKB, CCNA2, TPX2, and NCAPG, the pathological stage of ccRCC increased gradually. Compared with the individuals with low expression of AURKB, CCNA2, TPX2, and NCAPG, patients with high expression of AURKB, CCNA2, TPX2, and NCAPG have a poor overall survival.

Aurora kinase B (AURKB) is a serine/threonine kinase that participates in the regulation of chromosome arrangement and segregation by binding to microtubules [21]. Numerous studies have found that the overexpression of AURKB exists in a variety of cancer cell lines [22–24]. Sorrentino et al. found that AURKB is highly expressed in thyroid carcinoma, and its expression level is related to malignant degree. The block of AURKB expression or by using an inhibitor of Aurora kinase activity significantly reduced the growth of thyroid carcinoma cells [23]. Katayama et al. have similar findings in colorectal cancer [25], Smith et al. in lung cancer [22], and Chieffi et al. in prostate cancer [24]. Abnormal mitotic regulation can induce the production of aneuploid cells and act as a driving role in the process of malignant progression, while serine/theronine protein kinases of the Aurora family genes play a critical role in the regulation of key cell cycle processes. The abnormal expression of AURKB can produce malignant and invasive aneuploid cells. This further indicates that AURKB is related to tumorigenesis [26, 27]. With the discovery of abnormal expression of AURKB in cancer cells, researchers realized that it may become a new target for cancer treatments. At present, many AURKB inhibitors have been developed, including AZD1152, AT9283, VX-680/MK-0457, PHA-680632, AMG-900, PHA-739358, and CYC-116, and some of them have entered clinical trials [28].

The proteins encoded by CyclinA2 (CCNA2) belong to a highly conserved cyclin family, which promotes cell transformation by binding and activating cyclin-dependent kinases (CDKs) through G1/S and G2/M [29]. Previous studies have found that the overexpression of CyclinA2 occurs in lung cancer [30, 31], breast cancer [32, 33], colorectal cancer [34], and other tumors and related to poor prognosis of cancer patients. Aaltomaa et al. found that CyclinA2 was expressed in the cytoplasm of RCC but not in the normal tissue near the tumor, and the overexpression of CyclinA2 was related to the survival time of patients with RCC, suggesting that it may be a prognostic indicator of RCC [35]. The increase of the CyclinA2 expression is related to the uncontrolled and accelerated cell cycle, which leads to gene amplification and chromosome ectopia. Gopinathan et al. found that knockout CyclinA2 in mice can inhibit tumorigenesis [36]. Liang et al. found that the increased expression of sclerostin domain-containing protein1 (SOSTDC1) can inhibit CyclinA2, while SOSTDC1 can inhibit tumor growth [37]. CyclinA2 can not only be used as a predictor of prognosis and survival in patients with RCC but also has great potential in cancer treatment.

TPX2 microtubule nucleation factor (TPX2) encodes a microtubule-associated protein that activates cell cycle kinase called Aurora A and regulates mitotic spindles. The overexpression of TPX2 is related to the genesis of different cancers and is closely related to chromosome instability. The uncontrolled expression of TPX2 may eventually become the driving force of cancer development by inducing aneuploidy [38]. Zhang et al. found that compared with human bronchial epithelial cells (16HBE), TPX is overexpressed in malignant transformed 16HBE cells(16HBE-C) through anti-benzo[a]pyrene-trans-7,8-dihydrodiol-9,10-epoxide, in which TPX2 RNA interference (RNAi) can lead to S-phase arrest, inhibit cell proliferation, and induce cell apoptosis. TPX2 is tyrosine phosphorylated in malignant transformed 16HBE-C, and this phosphorylation may be involved in the malignant proliferation of cancer cells [39]. Ma et al. found that the level of the TPX2 protein in normal bronchial epithelium and alveoli was very low, while the level of TPX2 protein increased gradually in squamous metaplasia, dysplasia, and carcinoma in situ and invasive tumor. The immunohistochemical labeling index of TPX2 was related to the degree of differentiation, stage, and lymph node metastasis of lung squamous cell carcinoma, and the overexpression of TPX2 was significantly correlated with the decrease of 5-year survival rate [40]. Similar results were found in a variety of cancers, such as colorectal cancer [41], cervical cancer [42], and prostate cancer [43]. The expression of TPX2 in RCC was significantly higher than that in normal renal tissue, and it was related to tumor size, histological grade, tumor stage, and poor prognosis [44–47]. This may be due to the significant upregulation of TPX2 in RCC tissues, thus increasing the proliferation and invasive ability of renal cancer cells. From this point of view, TPX2 can not only become a target for RCC treatment but also play a role as an independent prognostic factor of RCC.

Non-SMC condensin I complex subunit G (NCAPG) coding a condensed protein complex subunit is responsible for chromosome condensation and stabilization during mitosis and meiosis [48]. In recent years, there are more and more studies on the abnormal expression of NCAPG in prostate cancer [49], lung cancer [50], breast cancer [51], and other cancers. In the study of Liu et al., NCAPG was found to be overexpressed in hepatocellular carcinoma compared with the adjacent normal tissue, and high levels of NCAPG expression were found to significantly correlate with recurrence, the time of recurrence, metastasis, differentiation, and TNM stage. The knockdown of NCAPG expression also inhibited tumor cell migration and the cell invasive capacity in vitro [52]. Through genome-wide functional knockout screen, Wang et al. believe that NCAPG is a necessary clinical-related target for the growth of hepatocellular carcinoma cells [53]. Ai et al. found that microRNA-181c (miR-181c) inhibits cancer by downregulating the expression of NCAPG, affecting the infiltration, migration, proliferation, and apoptosis of hepatoma cells [54]. In the study of Arai et al., microRNA-99a-3p downregulated the expression of NCAPG, thereby inhibiting cancer cell invasion in castration-resistant prostate cancer [49]. In conclusion, NCAPG represents a promising novel target and a prognostic biomarker for clinical management.

However, this study also has some shortcomings. Although the core genes screened in this study may play an important role in the occurrence of ccRCC, more clinical samples and patient prognosis information are still needed for verification.

## 5. Conclusion

To sum up, 251 differentially expressed genes and 10 hub genes (especially AURKB, CCNA2, TPX2, and NCAPG) were screened from ccRCC and normal renal tissues by microarray technology, which could be used as diagnostic and therapeutic biomarkers for ccRCC. AURKB, CCNA2, TPX2, and NCAPG which might be related to the occurrence and malignant progression of ccRCC.

## Figures and Tables

**Table 1:** Primers and their sequences for PCR analysis.

Primer	Sequence (5′–3′)
VEGFA-hF	GGCAACTTACTTAGCCTCTT
VEGFA-hR	AGGACAGTCTGAGTATGGGT
AURKB-hF	GTTCGCATTCAACCTACCT
AURKB-hR	GACGCCCAATCTCAAAGT
CCNA2-hF	AACTGGGATAAGGAAGCT
CCNA2-hR	CAGAAAGTATTGGGTAAGAA
MCM2-hF	TTCTCCCTCACTTGTCCC
MCM2-hR	CCTGTAATCCCAGCACTTT
MCM7-hF	GGGGTAGGCAGAACTCAA
MCM7-hR	CATGGAAGCGGTCTCAAA
SMC4-hF	AGTGGCGTAGCACAGTAA
SMC4-hR	ATTCCAAGATGATCCCTC
TPX2-hF	GCAATCCTTCTGCCTTAG
TPX2-hR	AGACCATCCTGGCTAACA
SLC2A1-hF	GAGACGGGAAACCATCAA
SLC2A1-hR	CTGCTCCTTCTTCAAACCAC
MCM5-hF	GGGTGCGAGGAGAACAGT
MCM5-hR	TGAGTCTGAGCCAGGGAG
NCAPG-hF	AGAGTATTGTTGGCTTCC
NCAPG-hR	AACTTCTGGACCATCACA

## Data Availability

The datasets used and/or analyzed during the current study are available from the corresponding author on reasonable request.
